# Implementing Three-Day Methadone in a Low-Threshold Bridge Clinic: Impact of OTP Referral Pathways on Treatment Retention

**DOI:** 10.21203/rs.3.rs-9088241/v1

**Published:** 2026-04-16

**Authors:** Sarah Rosenwohl-Mack, Leslie W. Suen, Ronald Hart, Scott Steiger, Jeffrey Hom, Hannah Snyder

**Affiliations:** 1 University of California, San Francisco, Department of Family and Community Medicine; San Francisco, CA, United States; 2 University of California, San Francisco, Division of General Internal Medicine; San Francisco, CA, United States; 3 University of California, San Francisco, Division of Substance Use and Addiction Medicine, Department of Psychiatry & Behavioral Sciences; San Francisco, CA, United States; 4 San Francisco Department of Public Health, San Francisco, CA, United States; 5 DrPH Program, Johns Hopkins Bloomberg School of Public Health, Baltimore, MD, United States

**Keywords:** Methadone, Opioid treatment program, Opioid use disorder, Bridge Clinic

## Abstract

**Background:**

Methadone is effective for treating opioid use disorder (OUD) but must be dispensed through federally regulated opioid treatment programs (OTPs). Geographic scarcity, waitlists, and limited intake schedules can delay OTP enrollment, causing patients to miss treatment opportunities. Regulations permit non-OTP practitioners to provide up to three days of methadone to bridge patients to OTP enrollment. This study evaluates the rates of linkage to and early retention in an OTP after initiation of three-day methadone at a Bridge Clinic.

**Methods:**

We conducted a retrospective case series of all patients who initiated methadone under the three-day rule at the San Francisco General Hospital Bridge Clinic between June 2024 and July 2025. The hospital also houses an OTP in the same building, which referred patients who were unable to complete intake. Outcomes included linkage to OTP care within seven days and retention at 30 days. Safety monitoring included review of all urine drug screens with either positive for methadone or negative for non-prescribed opioids, emergency department visits, hospitalizations within seven days, and Medical Examiner overdose reports. Multivariable logistic regression examined the association between OTP referral and 30-day retention.

**Results:**

Among 166 unique patients who received at least one dose of three-day methadone, 63% percent (104/166) linked to an OTP within seven days. Among patients who linked within seven days, 62% (64/104) were retained at 30 days. Referral from OTP to Bridge clinic was associated with 3.1-fold increased odds of 30-day retention. No methadone-related emergency department visits, hospitalizations, or deaths within 30 days of receipt of methadone were observed. Medical record review of unexpected urine drug screen results revealed that one patient was already enrolled in methadone treatment prior to receiving a dose.

**Conclusions:**

Initiating methadone in a low-threshold Bridge Clinic is feasible for engaging patients in OTP care. Referral from OTP to Bridge Clinic was associated with improved treatment retention.

Trial registration: not applicable

## BACKGROUND

The United States continues to face a devastating opioid overdose crisis, with approximately 79,000 opioid-involved overdose deaths in 2023.(1) Methadone, a full opioid agonist, is one of the most effective treatments for opioid use disorder (OUD), associated with approximately 50% reductions in both all-cause mortality and overdose deaths, as well as superior treatment retention compared to other medication options.(2,3) However, unlike buprenorphine, methadone for OUD cannot be prescribed and maintenance methadone must be dispensed through federally licensed Opioid Treatment Programs (OTPs). This creates unique barriers to access, including program waitlists and limited intake schedules.(4–8) Many patients face delays in accessing formal OTP enrollment due to lengthy intake processes and logistical barriers, potentially missing critical opportunities for treatment engagement.

Federal regulations permit non-OTP practitioners to provide up to 3 days of methadone through an exception commonly called the “The Three Day Rule” (Title 21, Code of Federal Regulations, Part 1306.07[b]), enabling immediate treatment initiation while patients transition to formal OTP enrollment.(9) This has been successfully applied across multiple healthcare settings. Bridge Clinic programs have reported positive outcomes, with a majority of patients successfully transitioning to OTPs after short-term methadone initiation.(10–12) body of evidence supports the viability of using the three-day rule to bridge patients to formal methadone treatment programs.

While these studies have demonstrated the feasibility of three-day methadone initiation, important gaps remain in the literature. Prior studies have not reported results of safety monitoring, such as a systematic review of urine drug screens to ensure appropriate patient selection or surveillance for adverse events after administration of methadone. Additionally, the impact of patient referral source on treatment engagement and retention remains unknown. This study aims to address these gaps by evaluating the effectiveness of a three-day methadone protocol at the Zuckerberg San Francisco General Hospital Bridge Clinic, with attention to comprehensive safety monitoring. A unique characteristic of this Bridge Clinic is that it is located in the same building as an OTP, and it received referrals if patients were unable to complete OTP intake. Accordingly, we also examined the association between direct OTP-to-Bridge Clinic referrals and 30-day treatment retention.

## METHODS

### Setting

The Zuckerberg San Francisco General Hospital Bridge Clinic is a low-threshold outpatient addiction medicine clinic with drop-in and scheduled availability. Patients can be referred or self-present. It is operated by the San Francisco Department of Public Health and the University of California, San Francisco. The clinic uses an interdisciplinary care model with addiction medicine physicians, nurses, a social worker, and navigators. The clinic provides treatment for opioid use disorder and all other substance use disorders. Services include same-day initiation of medications for opioid use disorder including buprenorphine and methadone, long-acting injectable medications, medications for other use disorders, contingency management, harm reduction counseling, linkage to community resources, and care coordination with other treatment programs. The clinic serves a marginalized population, with a high prevalence of individuals experiencing homelessness, co-occurring substance use, and mental health conditions. The clinic is in the same building as the Opiate Treatment Outpatient Program (OTOP), a licensed opioid treatment program that provides ongoing methadone maintenance treatment. This physical co-location facilitates warm handoffs and streamlined referrals between the two programs. Patients who are not referred from OTOP may access three-day methadone in many ways, including referral from the hospital or emergency department, referral from on-campus clinics that provide primary care or infectious disease testing and treatment, or self-presentation.

### Protocol

Bridge Clinic’s three-day methadone protocol was implemented under Title 21, Code of Federal Regulations, Part 1306.07(b), which permits non-OTP practitioners to administer or dispense up to three days of methadone to patients with OUD while arranging referral to formal OTP care. Adult patients (≥18 years) with moderate to severe OUD and current daily opioid use were eligible for treatment. Exclusion criteria included current enrollment in an OTP, clinical evidence of sedation or respiratory depression, known history of Torsades de Pointes, concern for low opioid tolerance, and refusal to provide urine drug screen or to sign release of information forms for OTP referral. Prior to the first dose, providers obtained a urine drug screen (UDS), which was sent to the laboratory with results available the next day), conducted a medical evaluation to confirm OUD diagnosis, and assessed for contraindications. Opioid withdrawal and results from urine drug screen were not required for initial dose. Methadone was administered as observed dosing in the clinic and documented in the medication administration record. Initial methadone doses were 30–40 mg on day one for patients using heroin or fentanyl, with an option for lower doses for patients using pills or if other concern for lower tolerance. Subsequent doses were increased by 10–20 mg based on ongoing withdrawal symptoms and clinical judgment, up to a maximum of 60 mg daily. Linkage to OTP care was initiated on the first day of treatment when feasible. Bridge Clinic staff contacted OTPs to arrange intake appointments and faxed clinical documentation including the provider note and signed release of information forms. Some patients were referred to Bridge Clinic directly from OTOP when they presented too late in the day for OTOP intake or when OTOP intake capacity was unavailable; these patients were prioritized for rapid return to OTOP once space became available.

### Study Design and Variables

We conducted a retrospective case series of all patients who initiated methadone under the three-day rule at the ZSFG Bridge Clinic between June 10, 2024, and July 30, 2025. Data were extracted from electronic health records (EHR, encompassing ZSFG hospital and Bridge Clinic), then matched to centralized records of San Francisco OTPs and records from the San Francisco Medical Examiner’s Office. An episode was defined as the complete three-day methadone treatment period inclusive of all visits. An encounter was defined as a single Bridge Clinic visit documented in the hospital EHR; thus, each episode could include up to three encounters. If a patient received multiple doses within a three-day period, this was counted as a single episode. Patients who accessed three-day methadone on multiple occasions were considered to have multiple episodes.

Patient characteristics included age, gender, race/ethnicity, primary language, insurance type (Medicare, Medicaid, private, or uninsured), and housing status (stable housing, unstable housing, or experiencing homelessness). The primary exposure of interest was referral source, specifically whether the patient was referred to Bridge Clinic from OTOP versus other sources (self-referral, emergency department, inpatient, or community referral). OTOP referral was defined as documentation of an OTOP visit without completed intake plus a same-day Bridge Clinic encounter for three-day methadone initiation. Treatment engagement outcomes included: (1) enrollment in any San Francisco OTP within one day after the last Bridge methadone dose (uninterrupted treatment); (2) enrollment within three days (primary linkage outcome); and (3) enrollment within seven days. The primary retention outcome was retention in OTP care at 30 days, which was defined as receiving at least 15 doses over the 30-day period following the last Bridge Clinic dose. Urine toxicology tested for amphetamines, cocaine, fentanyl, oxycodone, other opioids, benzodiazepines, and methadone.

### Safety Monitoring

Safety monitoring was conducted to ensure appropriate patient selection and identify adverse events. All UDS results were systematically reviewed by two authors (SRM, HS). For patients with UDS positive for methadone, we performed detailed chart reviews to assess if patients were already enrolled in another OTP and to determine the source of methadone exposure (recent medical treatment in hospital or emergency department settings, non-prescribed methadone use, or prior OTP enrollment). For patients with UDS negative for all non-prescribed opioids, we conducted chart reviews to confirm OUD diagnosis and clinical decision-making. Urine buprenorphine tests were not routinely collected. We reviewed all emergency department visits and hospitalizations occurring within seven days of any Bridge Clinic methadone dose to identify potential methadone-related adverse events, including oversedation, respiratory depression, cardiac events, or overdose. Medical Examiner records were reviewed to identify any deaths during the study period, including opioid-involved overdose deaths. We also reviewed encounters recorded as three-day methadone starts in which methadone was ultimately not administered, documenting reasons for nonadministration.

### Statistical Analysis

We calculated descriptive statistics for patient demographics, substance use patterns, treatment utilization, and clinical outcomes. Continuous variables are reported as means with standard deviations. Categorical variables are reported as frequencies and percentages. The primary analysis examined the association between OTOP referral and 30-day retention in OTP care using multivariable logistic regression. The model adjusted for age, race/ethnicity, gender, and housing status. Adjusted odds ratios with 95% confidence intervals and p-values are reported. Statistical significance was set at α = 0.05. Analyses were conducted using Stata v16.1 (StataCorp, College Station, TX). For patient characteristic tables, we used unique patients (first episode only) as the unit of analysis. For secondary analyses examining repeat utilization and episodes of care, we included all episodes. This study was deemed exempt by the University of California, San Francisco Institutional Review Board.

## RESULTS

Between June 10, 2024, and July 30, 2025, 166 unique patients initiated methadone under the three-day rule at the ZSFG Bridge Clinic, comprising 184 total episodes (Table 1).

The mean age was 42 years (SD 10). Two-thirds of patients were men (66%), 28% were women, and 5% identified as another gender. Most patients identified as White (63%), followed by Hispanic/Latino/a (16%), Black/African American (11%), Asian (4%), and other race/ethnicity (8%). Nearly all patients (96%) spoke English as their primary language, with 3% speaking Spanish. Most patients had Medicaid insurance (78%), with smaller proportions covered by Medicare (7%), private insurance (9%), or were uninsured (5%). Four in five patients (80%) were experiencing homelessness at the time of treatment initiation. Half of patients (50%) were referred to Bridge Clinic from OTOP, while the remainder were from other sources.

Urine drug screens were obtained from 152 patients (91%). The most common substances detected were fentanyl (83%), methamphetamine (71%), and cocaine (44%). Smaller proportions tested positive for benzodiazepines (10%) and heroin metabolites (7%).

Most patients (83%) had only one encounter during their first three-day methadone episode, while 16% had two encounters and 2% had three encounters. Twelve patients (7%) returned for a second episode of three-day methadone during the study period, with 5 patients (3%) returning for three or more episodes.

### Treatment Engagement and Retention

Among 166 unique patients receiving at least one dose of methadone at Bridge Clinic, 119 (72%) linked to a San Francisco OTP at any point during the study period (Table 2).

Over half (54%, 89/166) linked within three days of their last Bridge Clinic encounter, and 63% (104/166) linked within seven days. Among patients who successfully linked to OTP care, the vast majority (94%) enrolled at OTOP, the hospital-based OTP, with 6% enrolling at a communitybased private OTP in San Francisco. Retention at 30 days was 39% (64/166) for the overall cohort. Among patients who linked to an OTP within seven days, 30-day retention was 62% (64/104).

In multivariable logistic regression analysis restricted to the 104 patients who linked to an OTP within seven days, OTOP referral was associated with significantly higher odds of 30-day retention (aOR 3.1, 95% CI 1.5–6.3, p=0.002) (Table 3).

Experiencing homelessness was associated with lower odds of retention. Age, race/ethnicity, and gender were not significantly associated with retention in the adjusted model.

### Safety outcomes

Systematic review of all emergency department visits and hospitalizations occurring within seven days of Bridge Clinic methadone dosing identified zero visits that were related to methadone administration or overdose. Four patients (2%) died during the yearlong study period; none of these deaths were attributed to methadone.

During the first episode of three-day methadone, 37 patients had a UDS positive for methadone. Chart review of these cases revealed that 19 patients had documented recent methadone exposure from inpatient hospitalization, emergency department visits, or other OTPs in which they were no longer enrolled, with most of these exposures in the 2 weeks prior to dosing in Bridge Clinic. Ten patients endorsed recent use of non-prescribed methadone, and 7 patients had no clear documentation of methadone source, presumed to represent non-prescribed use. One patient was found to have been already enrolled in a San Francisco OTP. Three patients (2%) had UDS negative for all non-prescribed opioids; chart review confirmed that all three were appropriately selected for treatment as they were transitioning from buprenorphine to methadone.

Among 256 total encounters where methadone was considered, 16 encounters (6%) resulted in methadone not being administered. The most common reasons were sedation (n=5), followed by patient preference for buprenorphine (n=4), patients already or likely enrolled in an OTP (n=3), and patients leaving without being seen or leaving mid-visit (n=2). For two patients, the reason was not indicated in the chart.

## DISCUSSION

Our findings are consistent with prior work demonstrating the feasibility of using the three-day rule to initiate methadone in Bridge Clinic settings and achieve high rates of linkage to OTP care.(10–13) Over a 13-month period, 166 patients successfully initiated methadone under the three-day rule, with 63% linking to an OTP within seven days and 62% of those who linked remaining retained at 30 days. Direct referrals from OTOP to Bridge Clinic were associated with a three-fold increase in the odds of 30-day retention.

The three-fold increase in retention among patients referred from OTOP highlights the value of integrated care models linking OTPs and Bridge Clinics. Several mechanisms may explain this finding. Patients referred from OTOP had already pursued engagement with the OTP system, having presented for intake but encountering capacity limitations. The physical location of OTOP and Bridge Clinic in the same building facilitated warm handoffs and simplified logistics for patients returning to complete OTP enrollment. OTOP and Bridge staff were able to resolve barriers such as transportation difficulties and insurance issues early, rather than after patients had already disengaged from care. A nearby clinic that can offer three-day methadone may help engage patients in care who might otherwise be turned away if an OTP has reached its intake capacity. These findings have important policy implications, suggesting that health systems could consider incentivizing development of direct partnerships between OTPs and Bridge Clinics to improve access to methadone treatment.

Though this study was not powered to assess safety outcomes, there were no methadone-related emergency department visits, hospitalizations, or overdose deaths during the study period, despite serving a vulnerable population with high rates of polysubstance use. Care was provided by a large team of clinicians, and our findings suggest maintaining standardized safe practice was achieved. In 6% of encounters, methadone was not administered, most commonly due to sedation concerns, suggesting that clinicians were making clinical judgments to ensure patient safety. In 14 encounters, there were no urine drug screen results – in some of these cases, urine samples were obtained but not transported to the lab, while others weren’t collected at all, and this is an area of ongoing quality improvement. One patient was able to obtain a single dose of methadone at the Bridge Clinic despite being enrolled in an OTP, highlighting the potential risk of double dosing inherent to the clinical model. Notably, Bridge and OTOP clinic staff did identify this patient shortly thereafter, and usual state-mandated procedures for resolving registration in multiple OTPs were followed.

Several limitations should be considered when interpreting these findings. The retrospective design relied on chart review, and some data elements may have been incompletely documented. As a single-center study conducted in an urban academic medical center with an established Bridge Clinic and OTP in the same building, our findings may not be generalizable to settings without similar infrastructure or resources. We did not collect patient-reported outcomes, limiting our understanding of patient satisfaction and reasons for treatment discontinuation. Our definition of OTOP referral was based on same-day visits, which may have undercounted referrals if patients presented to OTOP and returned to Bridge Clinic on subsequent days. Finally, our retention measure was based on OTP dosing records that include take-home medications, which may overestimate true medication adherence if patients obtained but did not consume their doses. Although there were no methadone-related emergency department visits, hospitalizations, or overdose deaths during the study period, our study was not powered to detect rare events.

Future research should include prospective studies and qualitative research with patient interviews to better understand facilitators and barriers to treatment engagement and retention. Multi-site studies are needed to assess generalizability across different settings and populations’ and may increase the power to detect any adverse events. Cost-effectiveness analyses would inform resource allocation and policy decisions regarding the expansion of three-day methadone protocols. Finally’ studies examining optimal dosing protocols’ including evaluation of higher starting doses as explored in recent work following regulatory updates in 2024 (42 CFR Part 8)’ would further inform clinical practice.(13) At the XSFG Bridge Clinic’ we are currently implementing several protocol enhancements’ including increasing starting methadone doses and dispensing take home doses of methadone in addition to administering it per recent regulatory updates.

In our single-site retrospective analysis, implementing three-day methadone initiation in a low-threshold Bridge Clinic serving mostly patients experiencing homelessness facilitated linkage to OTP care, without evidence of adverse safety events. The impact of direct referrals from OTPs to Bridge Clinic highlights the value of integrated systems of care. The development of formal partnerships between OTPs and Bridge Clinics for patients who cannot complete same day OTP intakes may expand access to methadone treatment for individuals with opioid use disorder. This study adds to the growing literature on the feasibility of three-day methadone initiation from outpatient Bridge Clinics and linkage to formal OTP enrollment.

## Supplementary Material

Supplementary Files

This is a list of supplementary files associated with this preprint. Click to download.

- MethadonePatientInformation.pdf
- ThreeDayMethadoneProtocolfordissemination2025version.docx

## Figures and Tables

**Table 1.** Demographics and Outcomes of Patients Accessing Three-Day Methadone during 1^st^ Episode

Demographic	Participants (N=166)
**Age** (mean, SD)	42 years (SD 10)
**Gender**	
- Woman	47 (28%)
- Man	110 (66%)
- Other	9 (5%)
**Race/ethnicity**	
- Asian	6 (4%)
- Black/African American	18 (11%)
- Hispanic/Latino/a	26 (16%)
- White	103 (63%)
- Other	13 (8%)
**Language**	
- English	159 (96%)
- Spanish	5 (3%)
- Unknown	2 (1%)
**Primary Insurance**	
- Medicaid	130 (78%)
- Medicare	12 (7%)
- Private*	15 (9%)
- Uninsured	8 (5%)
- Jail	1 (1%)
**Person Experiencing Homelessness**	132 (80%)
**Referred from OTOP**	84 (50%)
**UDS completed during 1**^**st**^ **Episode**	152 (91%)
- Amphetamines	117 (71%)
- Methamphetamines	117 (71%)
- Barbiturates	0 (0%)
- Benzodiazepines	17 (10%)
- Cocaine	74 (44%)
- Fentanyl	137 (83%)
- Opiates	19 (11%)
- Oxycodone	5 (3%)
- Heroin	12 (7%)
- Tested negative for all opioids	3 (2%)
**Number of Encounters during 1**^**st**^ **Three-Day Methadone Episode**	
- 1 encounter	137 (83%)
- 2 encounters	26 (16%)
- 3 encounters	3 (2%)
**Deaths during study period**	4 (2%)
- Deaths within 30 days of seeing Bridge	1 (0.6%)
- Death from known overdose	0 (0%)

**Table 2.** Treatment engagement*

Treatment Engagement	Participants (N=166)
- Linked to SF OTP at all during study period	119
- Linked to OTP within 3 days of last three-day Bridge Encounter	89
- Linked to OTP in 7 days of last three-day Bridge Encounter	104
- OTOP	98 (94%)
- Community OTP	6 (6%)
- Still linked to OTP at 30 days**	64 (62%)

* Because data are by patient, only includes first episode (did not include repeat episodes for patients who pursued three-day methadone on multiple occasions)

**Table 3.** Odds of 30-Day Retention if Patient Referred from OTOP, adjusting for Age, Race/ethnicity, Gender, Housing Status, and UDS Negative for Opioids

	Adjusted Odds Ratio (95% CI)	p-value
**OTOP Referral**	3.1 (1.5 – 6.3)	0.002
**Age**	1.0 (0.97 – 1.03)	0.92
**Gender**		
Man	Ref	
Woman	0.9 (0.4 – 2.1)	0.82
Another Gender	1.5 (0.3 – 6.9)	0.59
**Race/ethnicity**		
Asian	Ref	
Black/African American	1.3 (0.2 – 9.9)	0.79
Latine	0.7 (0.1 – 4.9)	0.71
White	1.7 (0.3 – 10.4)	0.50
Other	0.5 (0.6 – 5.3)	0.60
**PEH**	0.4 (0.2 – 0.99)	0.05
**UDS Negative for non-Methadone Opioids**	7.1 (1.2 –40.8)	0.04

## Data Availability

The datasets used and/or analyzed during the current study are available from the corresponding author on reasonable request.

## List of abbreviations

EHR: Electronic health records
OUD: Opioid Use Disorder
OTP: Opioid Treatment Program
OTOP: Opiate Treatment Outpatient Program
UDS: Urine Drug Screen
